# COVID-19 and female reproductive health: pathophysiological mechanisms and clinical reproductive outcomes

**DOI:** 10.3389/fmed.2026.1911269

**Published:** 2026-09-11

**Authors:** Sandugash Yerkenova, Vyacheslav Lokshin, Gulim Aldangarova, Raikhan Skakova, Gaukhar Koshkimbayeva, Alla Mireeva, Lazzat Nurseitova, Nurzhamal Dzhardemaliyeva, Dana Akhmentayeva, Akmaral Maimakova, Bekzat Suieubekov, Sharapat Moiynbayeva

**Affiliations:** 1 Department of Science and Consulting, Kazakhstan Medical University “KSPH”, Almaty, Kazakhstan; 2 International Clinical Center for Reproductology “PERSONA”, Almaty, Kazakhstan; 3 Department of Obstetrics and Gynecology, Kazakhstan-Russian Medical University, Almaty, Kazakhstan; 4 A.N. Syzganov National Scientific Center of Surgery, Almaty, Kazakhstan; 5 Department of Obstetrics and Gynecology, Asfendiyarov Kazakh National Medical University, Almaty, Kazakhstan; 6 Department of General Practice No. 2, Asfendiyarov Kazakh National Medical University, Almaty, Kazakhstan; 7 Department of Internal Medicine, Asfendiyarov Kazakh National Medical University, Almaty, Kazakhstan; 8 Clinical Department of Medicine, University of International Business Named After Kenzhegali Sagadiev, Almaty, Kazakhstan; 9 Department of Clinical Diagnostical Laboratory, State Enterprise on the Right of Economic Management “Central City Clinical Hospital” of the Public Health Department, Almaty, Kazakhstan; 10 Department of Pediatric Anesthesiology and Intensive Care, S.D. Asfendiyarov Kazakh National Medical University, Almaty, Kazakhstan

**Keywords:** COVID-19, female reproductive health, fertility, long covid, ovarian reserve, preconception care, reproductive endocrinology

## Abstract

Coronavirus disease 2019 (COVID-19), caused by severe acute respiratory syndrome coronavirus 2 (SARS-CoV-2), has raised important concerns regarding its potential effects on female reproductive health. This review aimed to synthesize current evidence on the biological mechanisms, clinical manifestations, ovarian reserve, fertility potential, and assisted reproductive technology (ART) outcomes associated with COVID-19. A comprehensive literature search of PubMed/MEDLINE, Scopus, Web of Science, and Google Scholar was conducted for studies published between January 2020 and March 2026. Current evidence suggests that SARS-CoV-2 may affect female reproductive tissues through multiple interconnected pathways, including ACE2/TMPRSS2-mediated tissue susceptibility, immune dysregulation, oxidative stress, endothelial dysfunction, neuroendocrine disturbances, and potentially autoimmune mechanisms. Menstrual disturbances, such as changes in cycle length, menstrual flow, and dysmenorrhea, are among the most frequently reported reproductive manifestations, although most appear transient and reversible. Several studies have reported reductions in anti-Müllerian hormone (AMH) and hormonal alterations, particularly following severe infection; however, findings remain inconsistent. Importantly, most investigations demonstrate preserved ovarian reserve, fertility potential, and ART outcomes, including ovarian response, embryo development, clinical pregnancy, and live birth rates. Overall, current evidence suggests that COVID-19 may cause temporary reproductive alterations rather than permanent impairment of female fertility. Nevertheless, large prospective longitudinal studies are required to clarify long-term reproductive consequences and identify women at increased risk of persistent reproductive dysfunction.

## 1 Introduction

Since its emergence in late 2019, coronavirus disease 2019 (COVID-19), caused by severe acute respiratory syndrome coronavirus 2 (SARS-CoV-2), has affected hundreds of millions of individuals worldwide and generated substantial concerns regarding its long-term consequences for human health (1–3). Although the respiratory manifestations of COVID-19 were initially the primary focus of clinical investigation, it soon became evident that SARS-CoV-2 is a multisystem pathogen capable of affecting cardiovascular, neurological, endocrine, immune, and reproductive systems (1–4). Among women of reproductive age, increasing attention has been directed toward the potential impact of COVID-19 on reproductive health because reproductive function depends on the coordinated interaction of ovarian, endocrine, vascular, immune, and metabolic pathways that may be disrupted during and after infection (4–8). Consequently, understanding the short- and long-term reproductive consequences of COVID-19 has become an important priority for reproductive medicine, gynecology, and public health (5–8).

Biological evidence suggests several mechanisms through which SARS-CoV-2 may influence female reproductive function (4–10). Viral entry factors, including angiotensin-converting enzyme 2 (ACE2) and transmembrane serine protease 2 (TMPRSS2), are expressed in multiple reproductive tissues, including the ovary, endometrium, placenta, and hypothalamic-pituitary axis (9–12). In addition to potential direct tissue infection, SARS-CoV-2 may induce systemic inflammation, oxidative stress, endothelial dysfunction, neuroendocrine disturbances, and immune dysregulation, all of which could interfere with folliculogenesis, steroidogenesis, implantation, placental function, and reproductive hormone regulation (10–15). Experimental and clinical studies have demonstrated alterations in inflammatory cytokines, vascular homeostasis, and endocrine signaling following infection, supporting the biological plausibility of reproductive involvement (11–16). However, the relative contribution of these mechanisms and their clinical significance remain incompletely understood (12–16).

Accumulating clinical evidence has reported a wide spectrum of reproductive manifestations following COVID-19. Menstrual disturbances, including changes in cycle length, menstrual flow, dysmenorrhea, and menstrual regularity, have been frequently described in women recovering from infection (17–20). Similarly, several studies have reported alterations in ovarian reserve markers such as anti-Müllerian hormone (AMH), antral follicle count (AFC), follicle-stimulating hormone (FSH), and luteinizing hormone (LH) (17–19). Nevertheless, findings remain inconsistent. While some investigations have identified reductions in ovarian reserve markers and evidence of endocrine dysfunction, others have demonstrated preservation of ovarian function and normal reproductive outcomes (18, 19). These discrepancies may reflect differences in study design, disease severity, follow-up duration, patient populations, and methods of reproductive assessment.

Beyond menstrual and ovarian alterations, emerging evidence suggests that COVID-19 may also influence broader aspects of female urogenital and reproductive health through immune, endocrine, inflammatory, and healthcare-related pathways. Population-level studies from Kazakhstan have shown that the pandemic affected fertility dynamics, pregnancy-related outcomes, and the organization of maternal and primary healthcare services, indicating that reproductive consequences should be interpreted not only biologically but also within a public health and health-system context (20–22). At the clinical level, COVID-19 survivors may require preconception counseling and post-infection monitoring, as previous studies have reported pregnancy-planning concerns, menstrual disturbances, structural pelvic abnormalities, hormonal changes, and increased adverse outcomes among pregnant women with COVID-19 (21, 23, 24). In addition, women of reproductive age in Central Asia face a growing burden of cardiometabolic disorders, including cardiovascular disease, type 2 diabetes, and stroke, which may interact with reproductive health and increase vulnerability during and after infectious diseases (25). Finally, urinary tract infections represent an important component of female urogenital health, particularly among pregnant and postmenopausal women, because hormonal status, urinary physiology, microbiome disruption, catheterization, and antimicrobial resistance can complicate diagnosis and management (26). Therefore, the reproductive consequences of COVID-19 should be considered within a broader framework of women's urogenital and systemic health.

Although reproductive symptoms are increasingly recognized among women recovering from COVID-19 and in those with long COVID, the evidence remains fragmented and sometimes contradictory. Menstrual alterations, ovarian reserve changes, endocrine disturbances, and fertility-related concerns have been reported after SARS-CoV-2 infection, but it is still unclear whether these changes reflect direct viral effects on reproductive tissues, systemic inflammation, neuroendocrine stress responses, long COVID-related dysregulation, or pre-existing vulnerability (27). Moreover, reproductive symptoms may overlap with post-viral syndromes such as chronic fatigue syndrome and fibromyalgia, although systematic evaluation of hypothalamic-pituitary-gonadal axis function in these conditions has not consistently demonstrated clear gonadotropin or gonadal steroid abnormalities (28). Therefore, this review aims to synthesize current evidence on the effects of COVID-19 on female reproductive health by integrating mechanistic, clinical, endocrine, and fertility-related data. Specifically, we seek to clarify the biological pathways linking SARS-CoV-2 infection to reproductive dysfunction, summarize patterns of menstrual and hormonal disturbances, evaluate evidence on ovarian reserve and fertility potential, assess the relevance of long COVID and post-viral neuroendocrine mechanisms, and identify key knowledge gaps requiring future longitudinal and tissue-based studies.

## 2 Methods

A comprehensive narrative review with a structured literature search was conducted to identify and synthesize evidence regarding the effects of SARS-CoV-2 infection on female reproductive health. Electronic searches were performed in PubMed/MEDLINE, Scopus, Web of Science, and Google Scholar for studies published between January 2020 and March 2026. Search terms included combinations of the following keywords: “COVID-19”, “SARS-CoV-2”, “female reproductive health”, “ovary”, “ovarian reserve”, “anti-Müllerian hormone”, “AMH”, “antral follicle count”, “AFC”, “menstrual cycle”, “menstrual disorders”, “fertility”, “fecundability”, “assisted reproductive technology”, “ART”, “IVF”, “ICSI”, “pregnancy outcomes”, “placenta”, “endometrium”, “ACE2”, “TMPRSS2”, “oxidative stress”, “endothelial dysfunction”, “inflammation”, “long COVID”, and “reproductive recovery”.

**Figure 1:**
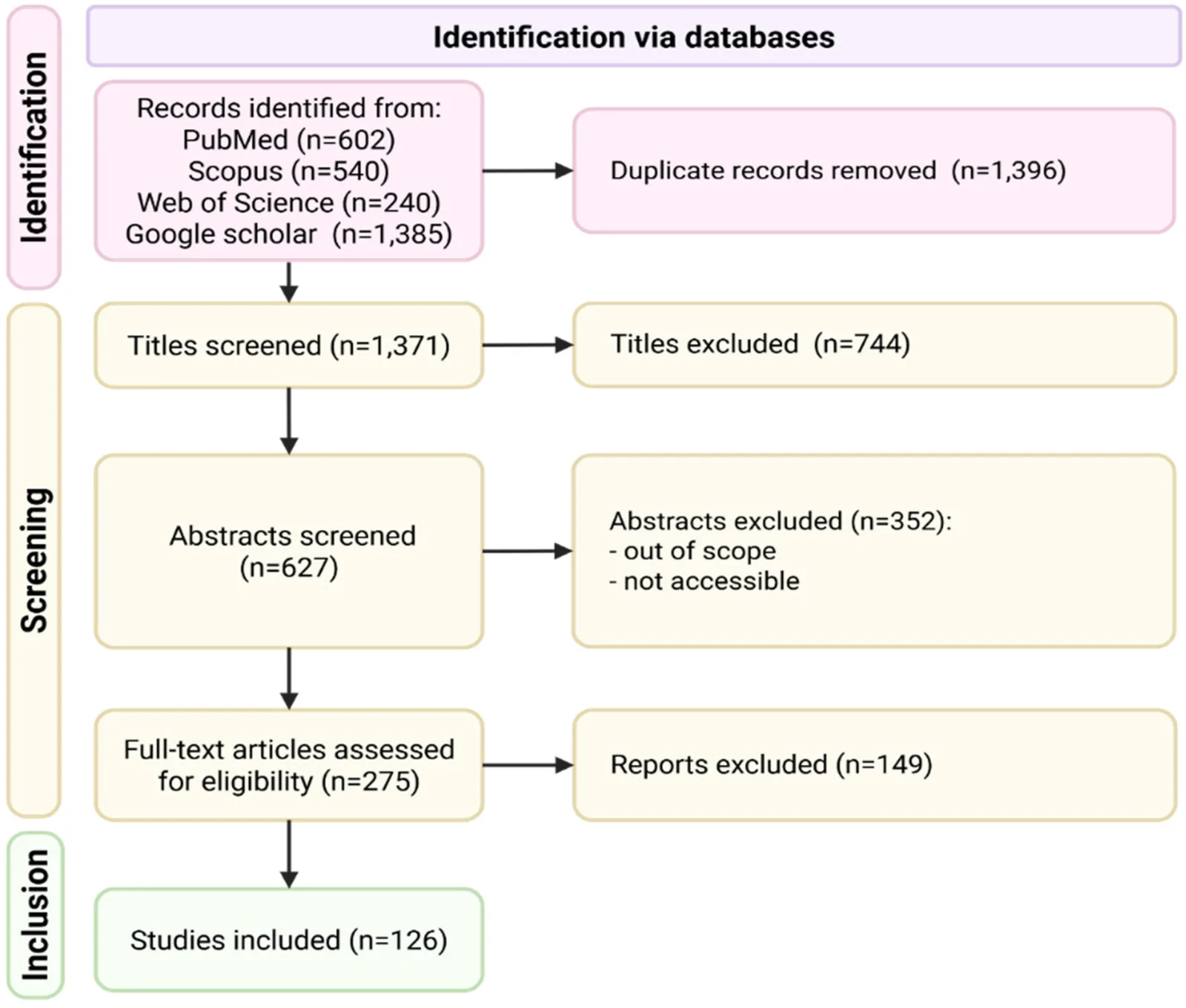
PRISMA flow diagram of the literature search and study selection process.

The study selection process is summarized in Figure 1. A total of 2,767 records were identified from PubMed (*n* = 602), Scopus (*n* = 540), Web of Science (*n* = 240), and Google Scholar (*n* = 1,385). After removal of duplicate records (*n* = 1,396), 1,371 titles were screened. Of these, 744 records were excluded at the title-screening stage. The abstracts of the remaining 627 records were reviewed, and 352 were excluded because they were outside the scope of the review or not accessible. Subsequently, 275 full-text articles were assessed for eligibility, of which 149 were excluded. Finally, 126 studies were included in the review. The excluded full-text articles were primarily removed because they did not address female reproductive outcomes, lacked sufficient outcome data, represented duplicate or overlapping populations, or did not meet the predefined eligibility criteria.

Eligible publications included original research articles, prospective and retrospective cohort studies, case-control studies, cross-sectional studies, registry analyses, systematic reviews, meta-analyses, and mechanistic experimental investigations relevant to female reproductive outcomes. Studies focusing exclusively on male reproductive health, animal models without direct relevance to female reproductive physiology, conference abstracts lacking sufficient methodological information, unavailable full texts, and duplicate reports were excluded.

Particular emphasis was placed on studies evaluating biological mechanisms of reproductive injury, menstrual and hormonal disturbances, ovarian reserve markers, fertility outcomes, assisted reproductive technology outcomes, and biomarkers of reproductive recovery. When multiple studies addressed the same outcome, priority was given to larger cohorts, prospective designs, systematic reviews, and studies providing quantitative reproductive endpoints. Evidence was critically evaluated according to study design, sample size, methodological quality, consistency of findings, and clinical applicability. Because this study was designed as a comprehensive narrative review with a structured literature search rather than a formal systematic review, formal risk-of-bias assessment and publication bias analysis were not performed. The collected evidence was then organized into thematic sections addressing biological mechanisms, clinical manifestations, ovarian function, fertility potential, reproductive recovery, and future research priorities. The literature search, study screening, eligibility assessment, and data extraction were independently performed by Sandugash Yerkenova and Sharapat Moiynbayeva. Any discrepancies were resolved through discussion and consensus.

## 3 Biological basis of sars-CoV-2 effects on the female reproductive system

**Figure 2:**
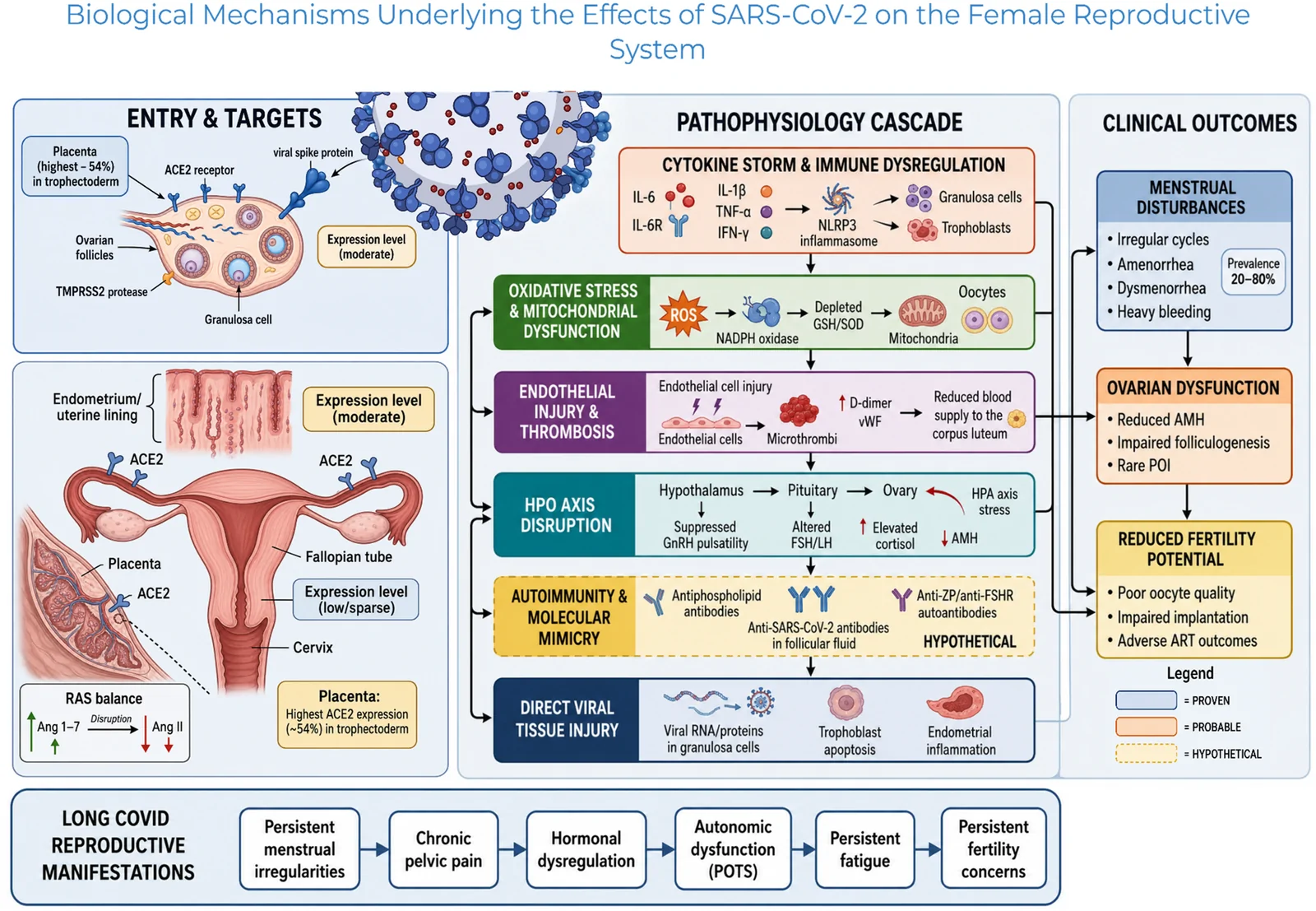
Biological pathways linking SARS-CoV-2 infection to female reproductive dysfunction. Proposed mechanisms through which SARS-CoV-2 may affect female reproductive health. Viral entry is mediated primarily by ACE2, TMPRSS2, and associated entry factors expressed in reproductive tissues, with the strongest evidence reported for placental trophoblasts and more variable expression observed in ovarian follicular cells and the endometrium (29–37). Following infection, SARS-CoV-2 may trigger multiple interconnected pathogenic pathways, including cytokine-mediated immune dysregulation (15, 31, 37–49), oxidative stress and mitochondrial dysfunction (50–56), endothelial injury and thrombosis (31, 38, 57–67), neuroendocrine disruption of the hypothalamic-pituitary-ovarian (HPO) axis (24, 45, 62, 68–75), autoimmunity and molecular mimicry (73, 76–81), and direct viral tissue injury (34, 37–39). These mechanisms may impair folliculogenesis, steroidogenesis, ovarian reserve, endometrial function, placental vascular integrity, and reproductive hormone regulation, ultimately contributing to menstrual disturbances, ovarian dysfunction, reduced fertility potential, adverse reproductive outcomes, and persistent post-COVID reproductive symptoms (45, 62, 71, 77, 82, 83). Solid boxes indicate mechanisms supported by substantial experimental or clinical evidence, whereas dashed boxes represent pathways that remain hypothetical or incompletely characterized. ACE2, angiotensin-converting enzyme 2; ADAM17, ADAM metallopeptidase domain 17; AMH, anti-Müllerian hormone; CTSL, cathepsin L; FSH, follicle-stimulating hormone; GnRH, gonadotropin-releasing hormone; HPO, hypothalamic-pituitary-ovarian; IL, interleukin; LH, luteinizing hormone; MCP-1, monocyte chemoattractant protein 1; NLRP3, NOD-like receptor family pyrin domain-containing 3; NRP-1, neuropilin-1; RAS, renin-angiotensin system; ROS, reactive oxygen species; SOD, superoxide dismutase; TMPRSS2, transmembrane serine protease 2; TNF-α, tumor necrosis factor alpha; vWF, von Willebrand factor.

Female reproductive dysfunction following SARS-CoV-2 infection appears to arise from multiple interconnected biological pathways rather than a single pathogenic mechanism. Current evidence supports the involvement of viral entry through ACE2- and TMPRSS2-expressing reproductive tissues, direct and indirect inflammatory injury, oxidative stress, endothelial dysfunction, neuroendocrine disturbances affecting the hypothalamic-pituitary-ovarian axis, and, potentially, autoimmune responses. The relative contribution and strength of evidence for these mechanisms vary considerably across reproductive tissues, with placental involvement being the most consistently demonstrated, whereas ovarian autoimmunity and long-term impairment of ovarian reserve remain less clearly established (Figure 2). These biological pathways may act independently or synergistically to influence menstrual function, ovarian reserve, fertility potential, and pregnancy outcomes.

### 3.1 ACE2/TMPRSS2 expression and reproductive tissue vulnerability to SARS-CoV-2

ACE2 and TMPRSS2, the principal host factors required for SARS-CoV-2 cellular entry, are expressed throughout the female reproductive system, although their distribution and abundance vary substantially among tissues. The placenta demonstrates the highest and most consistently documented expression of viral entry machinery, with ACE2 detected in 20%–63% of placental cell populations and reaching 54.4% positivity in trophectoderm cells, while TMPRSS2 expression ranges from 1.6% to 90.7% depending on trophoblast subtype (29). Functional studies have further confirmed the expression of ACE2, TMPRSS2, and furin in primary trophoblasts, supporting viral entry into placental cells (30, 31). In ovarian tissue, ACE2 and TMPRSS2 have been identified in granulosa and cumulus cells, and cultured follicular cells have been shown to support productive SARS-CoV-2 infection *in vitro* (32–35). However, transcriptomic analyses in women with polycystic ovary syndrome reported low or absent TMPRSS2 expression, suggesting that receptor abundance may be influenced by disease status, hormonal environment, and methodological differences between studies (36). Similarly, ACE2 and TMPRSS2 expression has been demonstrated in endometrial epithelial, stromal, and immune cells, including three-dimensional endometrial spheroid models capable of supporting viral infection and replication *in vitro* (8, 37, 84, 85). Although evidence for the fallopian tubes and cervix remains limited, available studies support the presence of viral entry factors throughout the female reproductive tract (86–88).

Beyond serving as a viral receptor, ACE2 is a key regulator of the renin-angiotensin system (RAS), which plays an important role in ovarian blood flow, follicular development, endometrial receptivity, and placental vascular function (89, 90). Binding of the SARS-CoV-2 spike protein may reduce ACE2 expression and activity, resulting in accumulation of angiotensin II and depletion of angiotensin-(1–7), thereby shifting the local tissue environment toward vasoconstriction, inflammation, oxidative stress, and fibrosis (31, 33). Such alterations may impair folliculogenesis, disrupt implantation processes, and compromise placental vascular homeostasis, even in the absence of extensive direct viral infection (90, 91). Therefore, reproductive dysfunction associated with COVID-19 is likely mediated by both tissue susceptibility to viral entry and secondary disturbances in ACE2-dependent signaling pathways that are essential for maintaining reproductive homeostasis.

### 3.2 Direct viral infection and tissue tropism in female reproductive organs

Direct evidence of SARS-CoV-2 tropism within female reproductive tissues remains considerably more limited than evidence for receptor expression, although several studies have demonstrated the capacity of reproductive cells to support viral infection. The strongest experimental evidence originates from primary human granulosa and cumulus cells isolated during IVF procedures, where SARS-CoV-2 infection resulted in intracellular viral RNA accumulation, expression of viral spike and nucleocapsid proteins, visualization of intact viral particles by transmission electron microscopy, and production of infectious progeny capable of infecting Vero E6 cells, thereby confirming completion of the viral replication cycle in ovarian follicular cells under *in vitro* conditions (34). Similarly, three-dimensional endometrial spheroid models support viral entry and replication and exhibit activation of inflammatory pathways, including increased IL-8 and MCP-1 production, particularly following decidualization (37). In contrast, the most convincing *in vivo* evidence has been reported in placental tissue, where viral RNA, proteins, and occasionally intact viral particles have been identified in syncytiotrophoblasts and cytotrophoblasts, frequently accompanied by trophoblast necrosis, chronic histiocytic intervillositis, villous inflammation, and placental vascular malperfusion (38, 39, 91). Placental infection has been associated with adverse obstetric outcomes, including fetal growth restriction, preterm birth, and stillbirth, particularly in severe maternal disease, although the frequency of placental involvement appears to vary according to maternal viral load, disease severity, and gestational timing of infection (38, 40).

Despite these observations, important uncertainties remain regarding the extent of direct reproductive tissue infection *in vivo*. Exposure of trophoblasts to SARS-CoV-2 spike protein alone induces inflammatory cytokine production, apoptosis, increased endothelial permeability, and activation of NF-κB signaling pathways, suggesting that placental dysfunction may occur even in the absence of extensive viral replication (31, 38). Furthermore, reproductive tissue susceptibility may be influenced by alternative viral entry mechanisms involving neuropilin-1, ADAM17, cathepsin L, and furin, which are expressed in placental tissue and may enhance infection when canonical ACE2/TMPRSS2 signaling is limited (32, 44). Interpretation of ovarian tropism is additionally complicated by the presence of the blood-follicle barrier, which may restrict viral access to granulosa and cumulus cells *in vivo* despite their demonstrated permissiveness under experimental conditions (41). Consistent with this possibility, studies examining follicular fluid and ovarian tissue from infected women have generally reported absent or very low viral detection rates, although sampling after recovery, limited cohort sizes, and methodological constraints may contribute to underestimation of transient infection events (42, 43). Collectively, current evidence supports proven placental infection *in vivo* and productive infection of ovarian follicular cells *in vitro* (34, 37), whereas endometrial infection and broader reproductive tract involvement remain biologically plausible but require further confirmation in well-designed clinical studies (38–44).

### 3.3 Immune dysregulation and cytokine-mediated reproductive damage

Immune dysregulation represents one of the most plausible mechanisms linking SARS-CoV-2 infection to reproductive dysfunction. Experimental studies have demonstrated that exposure of primary trophoblasts and trophoblast cell lines to SARS-CoV-2 spike protein induces a marked pro-inflammatory response characterized by increased production of IL-6, IL-8, TNF-α, and IFN-β, accompanied by activation of NF-κB signaling, enhanced expression of adhesion molecules, and induction of apoptotic pathways (31). These inflammatory changes are associated with reduced trophoblast syncytialization, decreased human chorionic gonadotropin production, and increased vascular permeability, all of which may compromise placental function (31). Notably, calcitriol partially attenuates these effects by suppressing ACE2/TMPRSS2 expression and inhibiting inflammatory signaling, providing further evidence that spike-mediated inflammation contributes directly to trophoblast dysfunction (33).

Similar inflammatory responses have been observed in endometrial models. Infection of three-dimensional endometrial spheroids results in increased secretion of MCP-1 and IL-8 together with dysregulation of innate immune pathways, including interferon signaling, complement activation, and Fc receptor-mediated responses (37). Decidualized spheroids exhibit a more pronounced inflammatory phenotype than non-decidualized models, suggesting that susceptibility to immune-mediated injury may vary according to endometrial status and reproductive stage (37). These findings indicate that SARS-CoV-2-associated inflammation may interfere with endometrial homeostasis, implantation processes, and early placental development through alterations in local cytokine networks and immune signaling pathways.

Evidence for cytokine-mediated ovarian dysfunction is less direct but supported by both mechanistic and clinical observations. COVID-19 has been associated with alterations in ovarian reserve and reproductive hormone profiles, including changes in AMH, FSH, and LH levels, as well as variable effects on oocyte yield and embryo quality in women undergoing assisted reproductive technologies (15, 45, 46). Potential mediators include activation of the NLRP3 inflammasome and increased production of pro-inflammatory cytokines such as IL-1β and IL-6, which may disrupt granulosa cell function, steroidogenesis, follicular development, and ovarian homeostasis (47, 48). Consistent with this hypothesis, granulosa cells obtained from women recovering from COVID-19 exhibit altered expression of genes involved in inflammation, cell-cycle regulation, vascular function, and gonadotropin receptor signaling (49). However, direct quantification of cytokine concentrations within follicular fluid, endometrial tissue, and other reproductive compartments remains limited, and current evidence is derived largely from experimental models, systemic inflammatory measurements, and indirect clinical associations (50). Consequently, cytokine-mediated reproductive damage is biologically plausible and supported by substantial mechanistic evidence, although tissue-specific inflammatory dynamics require further investigation.

### 3.4 Oxidative stress and mitochondrial dysfunction

Oxidative stress has emerged as a plausible mechanism linking SARS-CoV-2 infection to impaired female reproductive function. Direct evidence originates from follicular fluid analyses in women who had recovered from COVID-19 or received COVID-19 vaccination, which revealed significant alterations in redox homeostasis together with changes in inflammatory and metabolic profiles (50). Metabolomic assessment demonstrated disturbances in pathways related to energy metabolism, amino acid turnover, and lipid peroxidation, while biochemical analyses showed elevated oxidative stress markers and reduced antioxidant capacity compared with pre-pandemic controls (50, 51). These findings are particularly relevant because oocytes possess limited antioxidant defenses and high metabolic demands, rendering them highly susceptible to oxidative injury that may compromise meiotic competence, fertilization potential, and embryonic development (52).

Several mechanisms may contribute to excessive ROS generation in reproductive tissues during and after COVID-19. SARS-CoV-2 infection stimulates NADPH oxidase activity in immune and endothelial cells, while elevated concentrations of pro-inflammatory cytokines such as IL-6, TNF-α, and IL-1β further amplify oxidative stress through activation of inflammatory signaling pathways (51, 53). In addition, disruption of ACE2 signaling promotes accumulation of angiotensin II, a potent inducer of NADPH oxidase-mediated ROS production (89). Concurrent depletion of endogenous antioxidants, including glutathione, superoxide dismutase, and catalase, may further exacerbate oxidative injury (51, 54). Although direct evidence of ROS-induced oocyte damage in women with COVID-19 remains limited, the combination of altered follicular fluid redox balance, established oxidative stress pathways in COVID-19, and the recognized sensitivity of ovarian tissues to oxidative injury supports oxidative stress as a biologically plausible contributor to impaired fertility and reproductive dysfunction (45, 50–55).

Mitochondrial dysfunction represents a potential downstream consequence of oxidative stress and has been proposed as an additional mechanism of reproductive injury. Experimental studies in non-reproductive tissues indicate that SARS-CoV-2 proteins, particularly ORF9b, may localize to mitochondria, disrupt mitochondrial membrane potential, impair ATP production, and promote mitochondrial fragmentation, ultimately enhancing ROS generation and apoptotic signaling. Given the critical role of mitochondria in oocyte maturation, fertilization, and early embryonic development, such alterations could theoretically compromise reproductive competenc. However, direct evidence demonstrating mitochondrial localization of viral proteins, impaired mitochondrial function, or mitochondrial DNA damage in human oocytes following COVID-19 is currently lacking. Consequently, while mitochondrial dysfunction remains a compelling hypothesis, further studies employing ultrastructural, molecular, and functional analyses of reproductive tissues are required to determine its contribution to COVID-19-associated reproductive outcomes (56). In parallel, antioxidant-based interventions, including coenzyme Q10, N-acetylcysteine, vitamin C, and vitamin E, have been proposed as potential strategies to mitigate oxidative damage, although their efficacy specifically in women recovering from COVID-19 remains to be established in controlled clinical studies (47, 57).

### 3.5 Endothelial injury, vascular dysfunction, and thrombotic mechanisms

Endothelial dysfunction and vascular injury are central features of COVID-19 and may contribute substantially to reproductive complications (58, 59). The most convincing evidence has been reported in placental tissue, where exposure of trophoblasts to SARS-CoV-2 spike protein increases endothelial permeability, induces syncytiotrophoblast apoptosis, and promotes activation of procoagulant pathways through tissue factor expression and inflammatory signaling cascades (31). Consistent with these experimental observations, placentas from infected women frequently exhibit maternal and fetal vascular malperfusion, intervillous thrombi, villous stromal hemorrhage, and other vascular lesions associated with fetal growth restriction, preterm birth, and stillbirth (38, 60). Furthermore, several pathological features overlap with those observed in preeclampsia, including endothelial injury, impaired angiogenesis, and placental hypoperfusion, highlighting the importance of vascular dysfunction in COVID-19-associated pregnancy complications (31, 61).

A clinical study involving 158 women with post-COVID menstrual disorders identified endothelial dysfunction, microvascular impairment, and metabolic disturbances as potential contributors to abnormal menstrual patterns. Women who received an individualized pathogenetically based regimen incorporating hormonal, vascular-metabolic, neuroendocrine, and micronutrient support (including combinations of natural-estrogen hormonal therapy, L-arginine, quercetin, vitamin D3, melatonin, magnesium B6, folic acid, vitamin B12, and iron where clinically indicated) showed improved clinical outcomes compared with standard treatment protocols (62). Because ovarian follicular growth, ovulation, corpus luteum formation, and endometrial maturation are highly dependent on adequate vascular perfusion and angiogenesis, disruption of endothelial homeostasis may impair steroidogenesis, follicular development, and endometrial receptivity (63, 64). Supporting this hypothesis, elevated circulating markers of endothelial activation, including von Willebrand factor, soluble thrombomodulin, and circulating endothelial cells, have been documented during acute COVID-19 and may persist in patients with post-acute sequelae of SARS-CoV-2 infection (65–67).

Hypercoagulability and microthrombotic events provide an additional mechanism through which vascular injury may affect reproductive function. Severe COVID-19 is characterized by increased D-dimer, fibrinogen, factor VIII, and von Willebrand factor levels together with platelet activation and dysregulated fibrinolysis, creating a systemic prothrombotic state (58, 59, 69). Although direct evidence of microthrombi within ovarian or endometrial tissues remains limited, microvascular obstruction has the potential to compromise blood supply to developing follicles, the corpus luteum, and the endometrium, thereby impairing ovulation, luteal function, implantation, and early placental development (45, 55, 64). Clinical observations suggesting benefits from a pathogenetically based therapeutic strategy combining vascular-metabolic support (e.g., L-arginine and quercetin), hormonal therapy where indicated, and neuroendocrine and micronutrient supplementation in women with post-COVID menstrual abnormalities provide indirect support for this mechanism (62). Nevertheless, the reproductive consequences of systemic hypercoagulability remain incompletely defined, and future studies combining histopathological evaluation with advanced imaging approaches will be necessary to clarify the extent of microvascular injury within reproductive tissues (58, 59, 62, 68).

### 3.6 Neuroendocrine disruption and hypothalamic-pituitary-ovarian axis dysfunction

The hypothalamic-pituitary-ovarian (HPO) axis plays a central role in regulating menstrual cyclicity (69), ovulation, and reproductive hormone production and appears to be vulnerable to disruption following SARS-CoV-2 infection. Clinical studies consistently identify menstrual disturbances as one of the most frequently reported reproductive sequelae of COVID-19, with approximately 20%–30% of women experiencing alterations in cycle length, menstrual flow, or cycle regularity after infection (45, 46, 70, 71). Most abnormalities appear transient and resolve within several months, although persistent symptoms have been documented in a subset of patients (45, 46). Importantly, a randomized clinical study involving 158 women with post-COVID ovarian-endometrial dysregulative cycle disorders demonstrated that an individualized pathogenetically based treatment regimen combining hormonal, vascular-metabolic, neuroendocrine, and micronutrient support produced superior recovery of menstrual function and hormonal parameters compared with standard symptomatic treatment, supporting the existence of clinically relevant neuroendocrine alterations following COVID-19 (62).

Changes in reproductive hormone profiles provide additional evidence for HPO axis involvement. Several studies have reported transient reductions in AMH levels together with alterations in follicle-stimulating hormone (FSH) and LH concentrations following infection, although findings remain heterogeneous across cohorts (24, 45, 72). Current evidence suggests that these hormonal changes are influenced by infection severity, age, baseline ovarian reserve, and timing of assessment after recovery (45). While most women demonstrate normalization of hormonal markers over time, isolated cases of premature ovarian insufficiency have been reported, raising concerns regarding potential long-term reproductive consequences in susceptible individuals (73). Furthermore, transcriptomic analyses of granulosa cells from recovered women revealed persistent alterations in genes associated with inflammation, cell-cycle regulation, vascular homeostasis, and gonadotropin receptor signaling, suggesting that molecular changes may persist beyond the acute phase of infection (15, 49, 74, 75).

Systemic inflammation provides a biologically plausible mechanism linking COVID-19 to neuroendocrine dysfunction. Pro-inflammatory cytokines, including IL-1β, IL-6, and TNF-α, are known regulators of reproductive neuroendocrine function and can suppress GnRH neuronal activity, alter pituitary responsiveness to GnRH stimulation, and impair gonadotropin secretion (92–94). During acute COVID-19, circulating concentrations of these cytokines increase substantially, particularly in severe disease, creating conditions that may disrupt normal HPO axis signaling and activate the hypothalamic-pituitary-adrenal (HPA) axis, resulting in increased cortisol secretion. Elevated cortisol may, in turn, further amplify immune dysregulation by contributing to a sustained pro-inflammatory milieu and prolonged neuroendocrine dysfunction (95). Persistent low-grade inflammation observed in some patients after recovery may further contribute to prolonged reproductive symptoms (82, 83). Although direct measurements of GnRH pulsatility or pituitary responsiveness in women following COVID-19 are currently unavailable, the established effects of inflammatory mediators on reproductive neuroendocrinology provide a strong mechanistic framework for understanding infection-associated menstrual and hormonal disturbances (74, 75, 92–95).

Activation of the hypothalamic-pituitary-adrenal (HPA) axis represents another pathway through which COVID-19 may influence reproductive function. Acute illness, hospitalization, psychological stress, and prolonged recovery are all associated with increased cortisol secretion, which can suppress GnRH release and reduce pituitary sensitivity to reproductive hormonal signals (75). This mechanism resembles functional hypothalamic amenorrhea, a condition characterized by reversible suppression of reproductive function in response to physiological or psychological stressors (96). Supporting this concept, case reports have described women developing hypothalamic amenorrhea after COVID-19 with hormonal profiles consistent with hypogonadotropic hypogonadism, followed by gradual recovery after supportive management and resolution of stress-related factors (76, 96). These observations suggest that stress-mediated neuroendocrine suppression may contribute to transient reproductive dysfunction, particularly in women experiencing severe disease or prolonged recovery.

Neuroendocrine disruption may be further amplified by endocrine and systemic abnormalities associated with long COVID. Thyroid dysfunction, including both thyrotoxicosis and hypothyroidism, has been documented following SARS-CoV-2 infection and may independently contribute to menstrual irregularities, anovulation, and reduced fertility potential (70, 75, 76). In addition, women appear disproportionately affected by long COVID, and persistent symptoms such as fatigue, autonomic dysfunction, chronic inflammation, anxiety, and depression may create conditions that perpetuate HPO axis dysregulation (71, 77, 82, 83). Although the precise mechanisms remain incompletely understood, current evidence indicates that persistent reproductive symptoms after COVID-19 likely result from the combined effects of inflammatory, endocrine, metabolic, and neuroendocrine disturbances rather than a single isolated pathway. Longitudinal studies incorporating detailed hormonal profiling, autonomic assessment, and neuroendocrine monitoring will be essential to clarify the long-term reproductive consequences of SARS-CoV-2 infection (71, 82, 83, 96).

### 3.7 Autoimmunity and molecular mimicry in reproductive dysfunction

Autoimmune responses have emerged as a potential contributor to reproductive complications following SARS-CoV-2 infection. Among the most extensively studied autoimmune manifestations is the development of antiphospholipid antibodies (aPL), including anticardiolipin antibodies, anti-β2-glycoprotein I antibodies, and lupus anticoagulant, which have been detected particularly in patients with severe COVID-19 (78–80). Because these antibodies are established mediators of thrombosis, placental insufficiency, pregnancy loss, and preeclampsia, their appearance during COVID-19 has raised concerns regarding reproductive consequences (79). Reviews examining the relationship between COVID-19, fertility, and thrombosis suggest that aPL may act synergistically with the procoagulant environment already present during SARS-CoV-2 infection, thereby amplifying the risk of placental vascular complications and adverse pregnancy outcomes. Nevertheless, many COVID-19-associated aPL appear transient and do not fulfill diagnostic criteria for antiphospholipid syndrome, leaving their long-term clinical significance incompletely defined (79).

The presence of anti-SARS-CoV-2 antibodies within the ovarian microenvironment has generated additional interest regarding potential immune-mediated reproductive effects. Antibodies directed against viral antigens have been detected in follicular fluid following both natural infection and vaccination, indicating that humoral immune responses extend into the follicular compartment. Theoretical concerns have been raised regarding possible interactions between these antibodies and processes involved in oocyte maturation, fertilization, or early embryogenesis through immune complex formation and complement activation (45). However, available studies generally report no significant deterioration in assisted reproductive technology outcomes among women with detectable anti-SARS-CoV-2 antibodies in follicular fluid, suggesting that antibody presence alone does not necessarily imply pathogenicity (42, 45). At present, evidence supporting direct reproductive harm from anti-SARS-CoV-2 antibodies remains limited and largely speculative.

Molecular mimicry has been proposed as a mechanism through which SARS-CoV-2 could trigger autoimmune reactions targeting reproductive tissues. This process occurs when structural similarities between viral and host proteins lead to cross-reactive immune responses against self-antigens (76). Computational analyses have identified potential sequence homologies between SARS-CoV-2 proteins and proteins expressed in endocrine and reproductive tissues, raising the possibility of autoimmune targeting of ovarian structures (76). In particular, hypothetical cross-reactivity involving zona pellucida proteins, gonadotropin receptors, or other ovarian antigens has been suggested as a potential mechanism for post-infectious reproductive dysfunction (45). However, these hypotheses are currently supported primarily by bioinformatic and theoretical evidence, and experimental validation demonstrating pathogenic cross-reactive antibodies is lacking (76).

Rare reports of premature ovarian insufficiency following COVID-19 have further stimulated interest in autoimmune mechanisms. Case reports describe women developing amenorrhea, elevated FSH concentrations, and reduced estradiol levels after SARS-CoV-2 infection, findings compatible with ovarian insufficiency (73). Several mechanisms have been proposed to explain these observations, including autoimmune oophoritis, vascular injury, and inflammatory damage to ovarian tissue. Although such reports establish a temporal association between COVID-19 and premature ovarian insufficiency, they do not provide definitive evidence of causality, and autoimmune involvement remains unproven due to the absence of ovarian histopathology or validated ovarian autoantibody testing (73). Importantly, available data suggest that these cases are uncommon and do not indicate a widespread risk of ovarian failure among women recovering from COVID-19.

Overall, current evidence supports the existence of autoimmune activation following SARS-CoV-2 infection, but its specific role in female reproductive dysfunction remains incompletely understood. aPL represent the best-characterized autoimmune phenomenon with plausible reproductive consequences, whereas ovarian-specific autoimmunity and molecular mimicry remain largely hypothetical concepts requiring further validation (76, 79). Future investigations incorporating comprehensive autoantibody profiling, functional immunological assays, and long-term reproductive follow-up will be essential to determine whether autoimmune mechanisms contribute substantially to persistent reproductive symptoms after COVID-19 and to identify women at greatest risk for immune-mediated reproductive complications (73, 76, 78).

## 4 Integrated pathophysiological framework of COVID-19-induced reproductive dysfunction

Female reproductive dysfunction following SARS-CoV-2 infection is best understood as the consequence of multiple interconnected biological processes acting simultaneously rather than a single pathogenic pathway. The sequence begins with viral exposure and, in susceptible tissues, may involve direct infection of placental, ovarian, or endometrial cells expressing viral entry factors (29, 30, 34, 37). However, reproductive manifestations frequently occur without documented viral presence in reproductive organs, indicating that systemic consequences of COVID-19 play an equally important role (45). Following infection, inflammatory mediators, oxidative stress, vascular injury, neuroendocrine disturbances, and immune activation collectively create a biological environment capable of disrupting follicular development, endometrial function, placental homeostasis, and reproductive hormone regulation (31, 38, 51, 58, 74, 79, 82).

A defining feature of this model is the extensive crosstalk among pathological pathways. Systemic inflammation stimulates ROS generation, while oxidative stress amplifies inflammatory signaling through activation of NF-κB-dependent pathways, creating a self-reinforcing cycle of tissue injury (51, 82, 83). Simultaneously, oxidative stress contributes to endothelial dysfunction through reduced nitric oxide bioavailability and increased expression of vascular adhesion molecules, whereas endothelial injury further enhances ROS production through dysregulation of antioxidant systems and activation of NADPH oxidase (65). These interactions may explain why alterations in follicular fluid composition, endometrial physiology, and placental function can persist beyond the period of active infection (37, 38, 50).

Vascular and endocrine pathways further integrate systemic disease with reproductive outcomes. Endothelial dysfunction, hypercoagulability, and microvascular injury impair tissue perfusion and oxygen delivery, thereby affecting placental circulation, ovarian follicular development, corpus luteum function, and endometrial remodeling (38, 58–64). At the same time, inflammatory cytokines, activation of the hypothalamic-pituitary-adrenal axis, and endocrine disturbances alter reproductive hormone regulation through suppression of hypothalamic and pituitary signaling pathways (62, 74–76). The resulting hormonal imbalance contributes to menstrual irregularities, anovulation, and temporary reductions in reproductive function observed in many women following COVID-19 (45, 62, 70, 71, 96).

The relative importance of these mechanisms changes throughout the course of disease and recovery. During acute infection, systemic inflammation, stress responses, and direct viral effects predominate, whereas the recovery period is characterized by gradual normalization of hormonal, vascular, and metabolic processes (74, 75, 82, 83). Most women regain normal reproductive function within several months, consistent with the reversible nature of many inflammatory and neuroendocrine alterations (45, 46, 70, 71). In contrast, persistent symptoms associated with long COVID may reflect ongoing immune activation, chronic low-grade inflammation, autonomic dysfunction, endocrine abnormalities, or incomplete tissue recovery, thereby prolonging reproductive disturbances beyond the acute phase of infection (77, 82, 83).

Considerable interindividual variability exists in reproductive outcomes following COVID-19, suggesting that susceptibility is influenced by multiple host and disease-related factors. Greater infection severity, advanced reproductive age, reduced baseline ovarian reserve, pre-existing gynecological or metabolic disorders, genetic differences affecting immune and hormonal regulation, and variations in individual immune responses may all influence the extent and duration of reproductive dysfunction (45, 70, 72, 76, 78, 81). Differences in baseline reproductive and endocrine status may further contribute to this variability, with women presenting diminished ovarian reserve, pathological ovarian aging, endocrine disorders, or pre-existing immune dysregulation potentially exhibiting greater susceptibility to persistent reproductive abnormalities following SARS-CoV-2 infection (45, 76, 78, 81). Future studies should also investigate whether reproductive responses differ across distinct physiological stages of a woman's reproductive lifespan, including women with varying ovarian reserve, endocrine function, and hormonal milieu, to better define susceptible populations and improve individualized reproductive follow-up after COVID-19 (45, 76, 81). Psychological stress and social disruption associated with the pandemic may further exacerbate neuroendocrine dysregulation in susceptible individuals (77, 96). Collectively, current evidence supports a dynamic and multifactorial model in which interactions among inflammatory, oxidative, vascular, endocrine, and immune pathways determine both the severity and persistence of reproductive manifestations after SARS-CoV-2 infection.

## 5 Patterns and clinical characteristics of menstrual disturbances following COVID-19

Menstrual disturbances have emerged as one of the most frequently reported reproductive health consequences following SARS-CoV-2 infection. Evidence from large population-based cohorts, national surveys, and observational studies indicates that alterations in menstrual characteristics may occur across diverse populations of reproductive-age women, although the reported prevalence varies substantially because of differences in study design, outcome definitions, follow-up duration, and methods of menstrual assessment (97–104). Current evidence encompasses changes in cycle length, menstrual flow, dysmenorrhea, amenorrhea, and overall menstrual regularity, with most studies relying on self-reported outcomes and retrospective assessments (Table 1).

**Table 1:** Current evidence on menstrual disturbances associated with COVID-19.

Study	Country	Study design	Sample size	Study population (menstrual status)	Main findings	Limitations
Al-Najjar et al. (2022) (103)	Jordan and Iraq	Cross-sectional survey with pre/post comparison	*N* = 483	Currently menstruating women with previous COVID-19 infection	Changes in cycle length (47.2%), menstrual duration (41.8%), pain (42.0%), missed periods (22.6%), and intermenstrual bleeding (18.8%) were reported.	Selection bias; recall bias; no control group.
Issakov et al. (2022) (99)	Israel	National cross-sectional survey	*N* = 10,319 (7,476 vaccinated; 428 infected)	Currently menstruating premenopausal, non-pregnant women (≥18 years)	Menstrual disturbances were reported by 47.2% of infected women and 49.3% of vaccinated women (*p* = 0.387). Excessive bleeding accounted for 86.1% of abnormalities among infected women and 80.6% among vaccinated women, indicating a similar bleeding pattern in both groups.	Convenience sampling; recall bias; absence of uninfected controls.
Saxena et al. (2022) (98)	India	Retrospective follow-up (telephonic) pre/post study	*N* = 350	Currently menstruating women of reproductive age with previously regular menstrual cycles	Menstrual disturbances occurred in 59.1% of women after COVID-19, including altered menstrual flow (43.7%), irregular cycles (37.1%), worsening PMS (34.3%), and dysmenorrhea (22.4%). Most abnormalities resolved over time, with persistent irregularities reported by 17.1% at 1 year.	Single-center study; telephone recall bias; no uninfected control group; self-reported outcomes.
Alvergne et al. (2023) (97)	Multi-country (110 countries)	Retrospective cohort using prospectively tracked app-based cycle data	*N* = 6,514 (COVID *n* = 1,450; vaccinated *n* = 4,643; control *n* = 421)	Currently menstruating women aged 16–45 years with regular menstrual cycles	COVID-19 was associated with a transient increase in menstrual cycle length of 1.45 days (95% CI 0.86–2.04), compared with 1.14 days (95% CI 0.60–1.69) after vaccination. Cycle length returned to baseline in the subsequent cycle. Clinically significant changes (>8 days) occurred more frequently after COVID-19 (9.7%) than in controls (6.9%) or vaccinated women (6.3%).	Self-selection bias; app users may not represent the general population; COVID-19 status was self-reported; limited information on ethnicity, socioeconomic status, and gynecologic conditions.
Błażejewski and Witkoś (2023) (101)	Poland	Cross-sectional clinic-based study	*N* = 113	Currently menstruating women with previous COVID-19 infection	Menstrual disturbances occurred in 18.6% of women after COVID-19 and were associated with greater acute disease burden, long COVID symptoms, hospitalization, and obesity. Reported abnormalities decreased with increasing time since infection.	Small sample size; no control group; self-reported outcomes.
Singh et al. (2023) (102)	India	Hospital-based cross-sectional study	*N* = 132	Currently menstruating women aged 15–49 years with confirmed COVID-19	Menstrual abnormalities occurred in 22.0% of women after COVID-19, including prolonged cycles (14%), shortened cycles (8%), menstrual volume changes (23%), and increased dysmenorrhea (35%). Disturbances were more frequent in severe disease and resolved in most women within 1–2 months.	No pre-infection baseline assessment; no control group; hospital-based sample; self-reported outcomes; limited generalizability.
González et al. (2024) (104)	Spain	Retrospective observational cross-sectional study	*N* = 17,512 (infected subgroup *n* = 72)	Formerly menstruating women with previous SARS-CoV-2 infection	Menstrual disturbances occurred in 38.9% of infected women. Heavy bleeding and unexpected vaginal bleeding were the most frequent abnormalities.	Small infected subgroup; retrospective design; self-reported outcomes.
Jiang et al. (2025) (100)	China	Cross-sectional study	*N* = 869	Currently menstruating non-amenorrheic adult women (18–53 years)	Menstrual alterations were reported by 50.9% of women following SARS-CoV-2 infection. Delayed menstruation (19.7%), decreased menstrual volume (14.0%), increased menstrual volume (9.6%), prolonged menstruation (7.7%), worsened dysmenorrhea (7.2%), and advanced menstruation (4.5%) were the most common changes. Menstrual alterations were more frequent among women with chronic diseases (OR=2.60, 95% CI 1.33–5.08), fewer vaccine doses (OR=0.82 per additional dose, 95% CI 0.69–0.97), and a greater burden of acute and recovery-phase COVID-19 symptoms.	No uninfected control group; recall bias; causal relationships cannot be established.

CI, confidence interval; COVID-19, coronavirus disease 2019; OR, odds ratio; PMS, premenstrual syndrome; SARS-CoV-2, severe acute respiratory syndrome coronavirus 2.

The strongest available evidence originates from a multinational cohort using prospectively tracked menstrual cycle data from 6,514 women, which demonstrated a modest increase in cycle length following COVID-19 infection (+1.45 days; 95% CI 0.86–2.04) that returned to baseline in subsequent cycles (97). In contrast, survey-based and observational studies reported substantially higher frequencies of perceived menstrual disturbances, ranging from 38.9% to 59.1%, including irregular cycles, altered menstrual flow, dysmenorrhea, intermenstrual bleeding, and missed periods (98, 99, 103, 104). Importantly, longitudinal follow-up data suggest that most menstrual abnormalities are transient, with only 17.1% of women reporting persistent irregularities after one year (98). Collectively, these findings support an association between COVID-19 and temporary menstrual dysfunction; however, considerable uncertainty remains regarding the effects of disease severity, underlying gynecological conditions, and the long-term reproductive significance of these alterations (Table 1).

Hormonal disturbances have emerged as a potential mechanism linking COVID-19 with menstrual dysfunction and impaired reproductive health. However, the current evidence remains heterogeneous because studies differ substantially in design, disease severity, follow-up duration, and hormonal parameters evaluated (Table 2). Most investigations have focused on AMH as a marker of ovarian reserve (105–110), whereas comprehensive assessments of gonadotropins, sex steroids, thyroid hormones, and inflammatory biomarkers have been performed less frequently (24, 105, 108). Consequently, the evidence supporting alterations in ovarian reserve is stronger than that for broader hypothalamic-pituitary-ovarian axis dysfunction.

**Table 2:** Evidence on hormonal alterations and ovarian reserve.

Study	Country	Study design	Sample size	AMH findings	Other reproductive hormones	Additional findings	Key limitations
Ayar Madenli et al. (2022) (105)	Turkey	Prospective pre/post study	79	AMH decreased from 2.01 ± 0.70 to 1.49 ± 0.61 ng/mL (*p* < 0.001)	FSH increased (5.42 ± 1.71 to 6.06 ± 1.94 mIU/mL, *p* = 0.034); LH decreased (7.68 ± 4.30 to 5.22 ± 2.20 mIU/mL, *p* < 0.001); estradiol decreased (66.06 ± 58.34 to 53.30 ± 50.48 pg/mL, *p* = 0.016); prolactin, FT4, and TSH remained unchanged	Menstrual irregularities occurred in 42.0% of participants, and reduced menstrual flow was reported by 78.5%	Lack of a non-infected control group and short-term follow-up, limiting conclusions regarding persistence of endocrine alterations
Campitiello et al. (2023) (106)	Spain	Prospective ART cohort	46	No significant change in AMH (1.73 vs. 1.61 ng/mL; *p* = 0.789)	Not evaluated	No significant differences in AFC, ovarian response, oocyte yield, fertilization rate, or ART outcomes after infection	ART population may not represent the general reproductive-age population; limited endocrine assessment and short follow-up period
Doğan et al. (2024) (107)	Turkey	Prospective cohort	34	AMH decreased from 3.58 ± 1.51 to 3.27 ± 1.53 ng/mL (*p* = 0.025)	Not evaluated	AMH decline correlated with CRP (*r* = 0.542), ferritin (*r* = 0.570), and procalcitonin (*r* = 0.598), suggesting an association between systemic inflammation and reduced ovarian reserve	Small sample size, absence of an uninfected control group, and assessment restricted to AMH without broader endocrine profiling
Holovchak et al. (2024) (108)	Ukraine	Case-control study	100	Abnormal AMH levels were more frequent in post-COVID women than controls (25.0% vs. 10.0%, *p* < 0.05)	Abnormal FSH (22.5% vs. 5.0%), LH (36.3% vs. 15.0%), and progesterone (32.5% vs. 15.0%) were more common after COVID-19	Higher prevalence of menstrual disorders, luteal phase deficiency, and reduced ovarian function in women with prior COVID-19	Small control group, limited quantitative hormone reporting, and potential selection bias associated with reproductive clinic recruitment
Nazari et al. (2024) (109)	Iran	Case-control study	103	AMH was significantly lower in women recovering from severe COVID-19 than in controls (2.66 ± 1.95 vs. 3.38 ± 2.59 ng/mL; *p* = 0.03)	Not evaluated	Dysmenorrhea was significantly more frequent after severe COVID-19 (52.0% vs. 32.1%, *p* = 0.02)	Hormonal assessment was limited primarily to AMH, preventing comprehensive evaluation of hypothalamic-pituitary-ovarian axis function
Yerkenova et al. (2025) (24)	Kazakhstan	Case-control study	150	Lower AMH levels were observed in post-COVID women	Estradiol levels were higher, whereas TSH levels were lower in women with previous COVID-19 infection	Menstrual irregularities were more common after COVID-19 (36.0% vs. 20.0%, *p* = 0.035); ferritin levels were elevated and oophoritis was significantly more frequent (13.3% vs. 1.3%, *p* = 0.009)	Hormonal measurements were not synchronized to menstrual cycle phase, and longitudinal endocrine changes could not be evaluated

AFC, antral follicle count; AMH, anti-Müllerian hormone; ART, assisted reproductive technology; CRP, C-reactive protein; FSH, follicle-stimulating hormone; FT4, free thyroxine; LH, luteinizing hormone; TSH, thyroid-stimulating hormone.

Several studies reported evidence of impaired ovarian reserve after COVID-19. Doğan et al. observed a significant decline in AMH concentrations six months after infection, with the magnitude of AMH reduction correlating with CRP, ferritin, and procalcitonin levels, suggesting a relationship between ovarian dysfunction and systemic inflammation (107). Similarly, Ayar Madenli et al. demonstrated a significant decrease in AMH accompanied by increased FSH and reduced LH and estradiol concentrations three months after infection, indicating disruption of normal follicular development and endocrine regulation (105). Lower AMH levels were also reported in women recovering from severe COVID-19 compared with controls by Nazari et al. (109), while Holovchak et al. found higher frequencies of abnormal AMH, FSH, LH, and progesterone values among women with previous COVID-19 than among controls (108). In contrast, Campitiello et al. reported no significant differences in AMH, AFC, ovarian response, oocyte yield, or fertilization outcomes in women undergoing assisted reproductive treatment, suggesting that mild infection may have limited effects on short-term ovarian function (106). Furthermore, Yerkenova et al. identified lower AMH levels together with elevated estradiol, ferritin, and fibrinogen concentrations in women with a history of COVID-19, supporting the coexistence of endocrine and inflammatory alterations during recovery (24).

The endocrine abnormalities observed across studies parallel the menstrual disturbances described in post-COVID women. Reduced AMH levels were frequently accompanied by menstrual irregularities, decreased menstrual flow, dysmenorrhea, luteal phase deficiency, and impaired ovarian function (105, 108, 109). Holovchak et al. demonstrated that menstrual disorders and luteal phase deficiency were substantially more common among women with previous COVID-19 than among controls (108), whereas Ayar Madenli et al. reported menstrual irregularities in 42% of participants and reduced menstrual volume in 78.5% of women after infection (105). Likewise, Yerkenova et al. observed significantly higher rates of menstrual irregularities, elevated ferritin levels, reduced AMH concentrations, and an increased prevalence of oophoritis, suggesting that inflammatory, hormonal, and structural ovarian alterations may represent interconnected manifestations of post-COVID reproductive dysfunction (24). Nevertheless, interpretation of these findings should be cautious because most studies were limited by small sample sizes, absence of pre-infection hormonal measurements, lack of cycle-synchronized hormone testing, and relatively short follow-up periods (105–110). Therefore, although current evidence supports an association between COVID-19, endocrine alterations, and menstrual dysfunction, the persistence and clinical significance of these abnormalities remain uncertain and require confirmation in large prospective longitudinal studies.

Overall, the available evidence suggests that COVID-19 may induce transient hormonal disturbances in a subset of reproductive-age women, particularly those with severe disease, pronounced inflammatory responses, or clinically significant menstrual abnormalities (105, 107–109). The most consistent finding across studies is a reduction in AMH levels, whereas alterations in FSH, LH, estradiol, progesterone, and thyroid hormones have been reported less consistently (24, 105, 107, 108). Importantly, the discordant findings between studies demonstrating impaired ovarian reserve and those showing preserved ovarian function and ART outcomes indicate that endocrine consequences may depend on disease severity, baseline reproductive status, and the timing of hormonal assessment (106, 109). Collectively, current evidence supports a potential association between COVID-19 and temporary disruption of ovarian and endocrine function but remains insufficient to conclude that SARS-CoV-2 infection causes permanent impairment of ovarian reserve or long-term reproductive capacity (24, 105–110).

## 6 Ovarian function, ovarian reserve, and fertility potential

Ovarian function and ovarian reserve are critical determinants of female reproductive potential and provide important insight into the possible long-term reproductive consequences of COVID-19 (16, 111, 112). Current evidence primarily relies on surrogate markers such as AMH and AFC, which reflect the quantity of the remaining follicular pool and predict reproductive capacity. However, available studies have reported conflicting results. Several investigations demonstrated reductions in AMH concentrations and, in some cases, decreases in AFC following SARS-CoV-2 infection, suggesting a potential adverse effect on ovarian reserve (107, 109, 111). In a multicenter prospective study, Gullo et al. reported a 27.4% reduction in AMH accompanied by a decline in AFC after infection, whereas Doğan et al. and Nazari et al. also observed significantly lower AMH levels during the recovery period, particularly among women with more severe disease (107, 109, 111). Moreover, the association between AMH decline and inflammatory markers such as CRP, ferritin, and procalcitonin suggests that ovarian impairment may be linked to systemic inflammatory responses rather than direct viral injury alone (107).

In contrast, several studies failed to demonstrate clinically significant deterioration in ovarian reserve. A modest reduction in AMH following infection did not reach statistical significance, and no significant differences were observed in AMH or AFC among women undergoing assisted reproductive treatment (16, 112). Consistent with these findings, a recent systematic review concluded that the available evidence remains inconclusive, with only a minority of studies demonstrating measurable reductions in ovarian reserve markers and most AMH values remaining within normal reproductive ranges (113). Furthermore, current data do not support a detrimental effect of COVID-19 vaccination on AMH or AFC (113).

**Figure 3:**
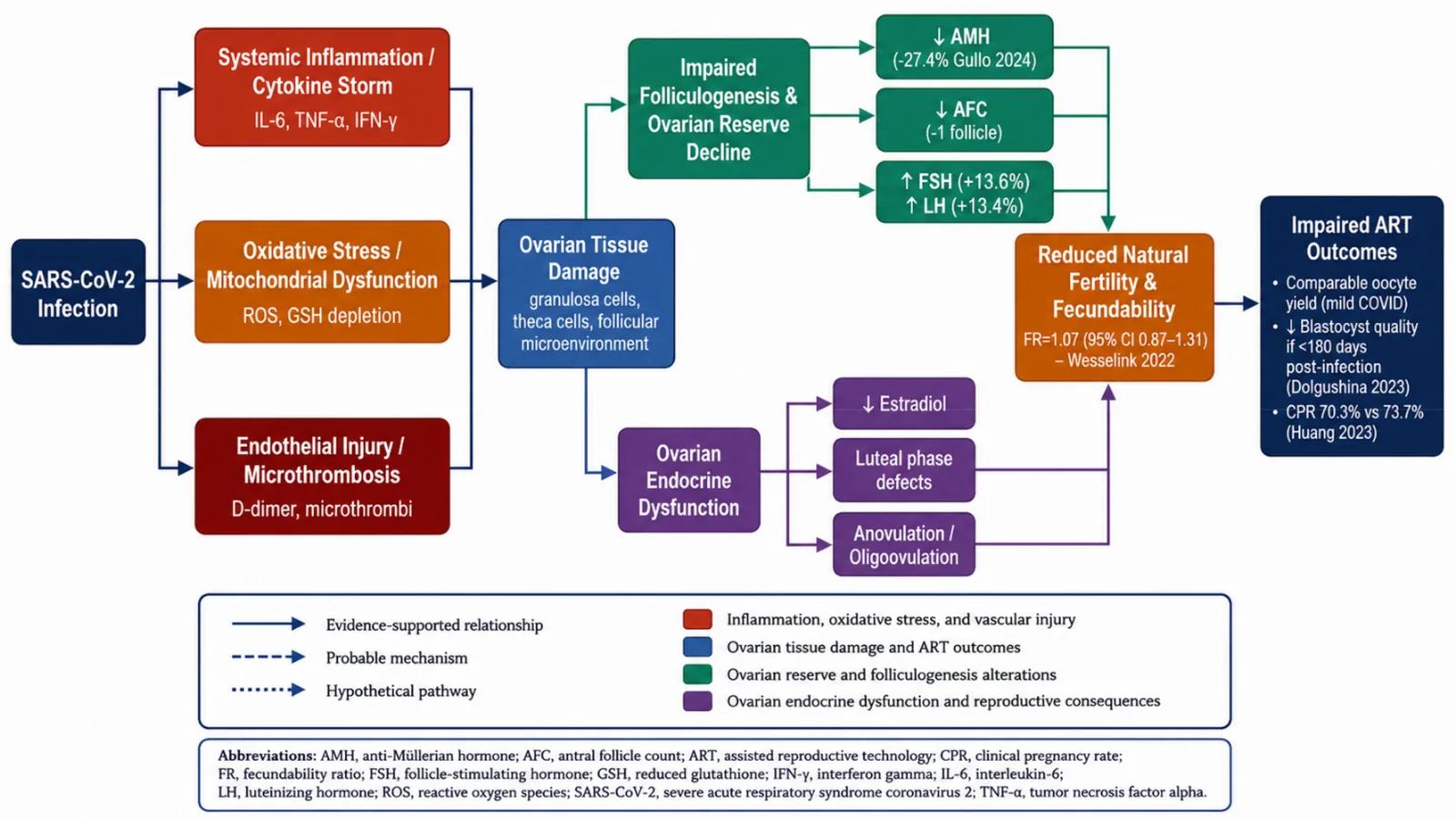
Proposed mechanisms linking COVID-19 to ovarian reserve, ovarian function, fertility potential, and ART outcomes: SARS-CoV-2 infection may affect female reproductive health through systemic inflammation, oxidative stress, mitochondrial dysfunction, and endothelial injury, leading to ovarian tissue damage and alterations in folliculogenesis, ovarian reserve, and ovarian endocrine function (107, 109, 111). Proposed downstream effects include reductions in anti-Müllerian hormone (AMH) and antral follicle count (AFC), changes in gonadotropin and sex steroid regulation, ovulatory dysfunction, and potential effects on fertility potential and assisted reproductive technology (ART) outcomes (107, 109, 111, 114–122). Although transient alterations in ovarian reserve markers and endocrine parameters have been reported, current evidence indicates that fecundability, ovarian response, embryo development, clinical pregnancy, and live birth outcomes are generally preserved following recovery from COVID-19 (114, 116, 118–122). Within the figure, FSH denotes follicle-stimulating hormone, LH denotes luteinizing hormone, ROS denotes reactive oxygen species, GSH denotes reduced glutathione, CPR denotes clinical pregnancy rate, and FR denotes fecundability ratio. Solid arrows indicate relationships supported by available clinical evidence, dashed arrows indicate probable mechanisms, and dotted arrows indicate hypothetical pathways requiring further investigation. Red and orange boxes represent inflammatory, oxidative stress, and vascular injury pathways; blue boxes represent ovarian tissue damage and ART-related outcomes; green boxes represent ovarian reserve and folliculogenesis alterations; purple boxes represent ovarian endocrine dysfunction and its reproductive consequences.

Taken together, the available evidence suggests that ovarian reserve may be transiently affected in a subset of women following COVID-19, particularly those with severe disease or pronounced inflammatory responses. However, the overall effect appears modest, and current studies do not provide convincing evidence of permanent follicular depletion or accelerated ovarian aging. Interpretation remains limited by heterogeneous study populations, differences in disease severity, variable follow-up periods, and inconsistent assessment of ovarian reserve parameters. Consequently, larger longitudinal studies incorporating serial AMH, AFC, and reproductive outcome measurements are needed to determine whether the observed changes represent temporary physiological responses or clinically meaningful reductions in long-term fertility potential (Figure 3) (16, 107, 111–113).

Evidence regarding ovarian endocrine function after COVID-19 is considerably less consistent than that for ovarian reserve. Unlike AMH, which reflects the size of the follicular pool, gonadotropins and sex steroids provide insight into hypothalamic-pituitary-ovarian axis activity and ovarian responsiveness. Several studies reported alterations in endocrine parameters following SARS-CoV-2 infection, whereas others found no measurable hormonal changes, suggesting that endocrine effects may depend on disease severity, timing of assessment, and underlying reproductive status (24, 111, 112, 114, 115).

Among studies reporting endocrine abnormalities, Gullo et al. observed post-infection increases in FSH and LH concentrations without significant changes in estradiol levels, suggesting compensatory pituitary stimulation despite preserved ovarian steroidogenesis (111). Similarly, Küçükyurt et al. reported post-infection elevations in FSH, LH, estradiol, and prolactin, although the lack of detailed quantitative data limits interpretation of these findings (110). Ayar Madenli et al. documented a different hormonal pattern characterized by reduced estradiol and LH concentrations three months after infection, which may indicate transient impairment of follicular activity and ovarian endocrine function during recovery (105). In contrast, Yerkenova et al. identified higher estradiol levels together with lower AMH concentrations in women with previous COVID-19, suggesting that endocrine alterations may coexist with inflammatory and structural reproductive abnormalities rather than reflecting a uniform pattern of ovarian dysfunction (24).

However, several investigations failed to detect significant endocrine disturbances. Herrero et al. reported that ovarian function parameters in ART patients largely normalized between 9 and 18 months after mild COVID-19 infection, with VEGF levels recovering over time and no persistent impairment of ovarian response observed during follow-up (114). Similarly, Soysal et al. and Kahyaoglu et al. found no significant changes in early-follicular gonadotropins, estradiol concentrations, or ovarian reserve markers following COVID-19 recovery (112, 115). These negative findings are particularly important because they suggest that endocrine homeostasis remains preserved in many women, especially after mild infection. Taken together, the available evidence does not support a consistent endocrine signature following COVID-19. Instead, current findings suggest that ovarian endocrine disturbances, when present, are likely transient, heterogeneous, and potentially influenced by inflammatory burden, disease severity, and individual reproductive characteristics. Further longitudinal studies incorporating standardized cycle-phase hormone measurements and ovulatory assessments are required to determine the clinical significance and duration of these endocrine changes (24, 105, 110–112, 114, 115).

Ovarian function, ovarian reserve, and fertility potential represent interconnected aspects of female reproductive health that may be affected by SARS-CoV-2 infection. Current evidence suggests that although COVID-19 can be associated with measurable alterations in ovarian physiology, the magnitude and persistence of these effects remain uncertain. Studies evaluating ovarian reserve have reported conflicting findings. Several investigations demonstrated reductions in AMH concentrations following infection, with some studies also documenting decreases in AFC, suggesting a potential reduction in follicular reserve (107, 109, 111). In particular, Gullo et al. reported a 27.4% decline in AMH together with a reduction in AFC, whereas Doğan et al. observed significant AMH decreases that correlated with inflammatory markers including CRP, ferritin, and procalcitonin (107, 111). Similarly, Nazari et al. found lower AMH levels among women recovering from severe COVID-19 compared with controls, indicating that disease severity may influence ovarian reserve outcomes (109).

Evidence regarding ovarian function is similarly heterogeneous. Some studies reported alterations in gonadotropins and sex steroids following infection, including increased FSH and LH concentrations (111), reduced estradiol and LH levels (105), or broader endocrine abnormalities involving FSH, LH, estradiol, prolactin, and progesterone (9, 24, 105, 110). However, other cohorts found no significant differences in ovarian endocrine parameters before and after infection (112, 114, 115). Importantly, the observed endocrine changes do not follow a consistent pattern across studies, suggesting that ovarian dysfunction is unlikely to be a universal consequence of COVID-19. Instead, endocrine disturbances appear to occur predominantly in selected subgroups, particularly women with severe disease, elevated inflammatory burden, or pre-existing reproductive vulnerability (107, 109, 110).

Despite concerns regarding ovarian reserve and endocrine function, current evidence provides limited support for a substantial reduction in fertility potential. The largest prospective preconception cohort reported no meaningful association between female SARS-CoV-2 infection and fecundability, with conception probabilities remaining comparable between infected and non-infected women (116). Likewise, most studies evaluating assisted reproductive technology outcomes found no significant adverse effects on ovarian response, oocyte yield, fertilization rates, embryo development, or clinical pregnancy outcomes following recovery from COVID-19 (114). Although some reports suggest that disease severity, inflammatory status, or timing of conception attempts relative to infection may influence specific reproductive outcomes (107, 109), the overall body of evidence indicates that reproductive capacity is generally preserved in most women after recovery. Consequently, while transient disturbances in ovarian reserve markers and endocrine function may occur, current data do not support widespread or permanent impairment of female fertility potential following COVID-19 (16, 111–116).

Overall, current evidence suggests that prior SARS-CoV-2 infection has minimal impact on IVF and ICSI outcomes, with most studies reporting preserved ovarian response, fertilization capacity, embryo development, and pregnancy success rates following recovery from COVID-19 (Table 3) (42, 118–122). Prospective and retrospective cohort studies consistently demonstrated no significant differences in oocyte yield, fertilization rates, embryo quality, or clinical pregnancy rates between previously infected and uninfected women. For example, Huang et al. reported comparable oocyte retrieval (11.4 vs. 11.6), fertilization rates (66.3% vs. 64.8%), blastocyst formation rates (72.6% vs. 72.2%), and clinical pregnancy rates (70.3% vs. 73.7%) between infected and control groups (118). Similarly, Youngster et al. observed no significant differences in mature oocyte yield, embryo quality, or pregnancy outcomes, although a decline in oocyte yield was noted when oocyte retrieval occurred more than 180 days after infection (119). Similarly, Dolgushina et al. found no significant differences in ovarian response, embryogenesis parameters, pregnancy, or live birth outcomes between women with and without previous COVID-19. However, a higher proportion of poor-quality blastocysts was observed when oocyte retrieval was performed within 180 days after infection, and moderate COVID-19 was associated with an increased risk of early miscarriage (42).

**Table 3:** IVF and ICSI outcomes following SARS-CoV-2 infection and COVID-19 vaccination.

Study	Country	Study design	Population	Oocyte yield	Fertilization rate	Embryo quality	Clinical pregnancy rate	Live birth rate	Main findings
Aharon et al. (2022) (122)	USA	Retrospective cohort	COH: 222 vaccinated vs 983 unvaccinated; FET: 214 vaccinated vs 733 unvaccinated	No difference; adjusted β = 0.01 ± 0.57; *p* = 0.99	80.7% vs 78.7%; adjusted β = 0.02 ± 0.02; *p* = 0.20	No effect on blastulation (β=0.02 ± 0.02; *p* = 0.27) or euploid embryo rate (β=0.05 ± 0.03; *p* = 0.08)	59.5% vs 63.7%; adjusted aOR = 0.79 (95% CI 0.54–1.16)	Ongoing pregnancy: aOR = 0.90 (95% CI 0.61–1.31)	COVID-19 vaccination showed no adverse effects on ovarian stimulation, embryo development, implantation, or pregnancy outcomes.
Youngster et al. (2022) (119)	Israel	Retrospective matched cohort	121 infected women vs 121 matched controls	12.50 vs 11.29; *p* = 0.169	Mature oocyte rate: 78% vs 82%; *p* = 0.144	No significant differences in good-quality embryos, frozen embryos, or fertilization outcomes	43% vs 40%; *p* = 0.737	Not reported	Overall IVF outcomes were unchanged, although oocyte yield decreased when retrieval occurred >180 days after infection.
Dolgushina et al. (2023) (42)	Russia	Prospective observational cohort	240 infertile women: 135 with prior COVID-19 (85 mild, 50 moderate) vs 105 controls	No significant differences reported	No significant differences reported	Overall embryogenesis parameters were comparable; however, ART within ≤180 days after infection was associated with a higher proportion of poor-quality blastocysts (*p* = 0.006)	Overall pregnancy outcomes comparable	Not reported	ART outcomes were generally comparable; severe disease and shorter post-infection intervals may adversely affect blastocyst quality.
Huang et al. (2023) (118)	China	Prospective cohort	451 women: 252 prior COVID-19 vs 199 controls	11.4 ± 8.3 vs 11.6 ± 7.7; *p* = 0.457	66.3 ± 24.4% vs 64.8 ± 24.2%; *p* = 0.463	Good-quality embryo rate: 28.6 ± 26.5% vs 27.6 ± 25.8%; *p* = 0.681; blastocyst formation rate: 72.6 ± 31.3% vs 72.2 ± 27.0%; *p* = 0.322	70.3% vs 73.7%; *p* = 0.590	Not reported	No significant effects of prior COVID-19 on ovarian response, embryo development, implantation, or clinical pregnancy outcomes.
Yan et al. (2025) (120)	China	Self-controlled repeated-cycle study	66 women undergoing IVF/ICSI before and after COVID-19 infection	12.41 ± 6.90 vs 14.26 ± 8.49; *p* = 0.172	72.62 ± 20.18% vs 71.24 ± 22.88%; *p* = 0.714	No significant differences in embryo yield, blastocyst formation, or high-quality embryo rates; MII/oocyte rate decreased (88.2% vs 76.9%; *p* = 0.006)	63.3% vs 57.6%; *p* = 0.883	No significant differences in miscarriage, preterm birth, or full-term delivery outcomes	No clinically significant changes in ovarian response, embryo quality, or pregnancy outcomes following mild COVID-19.
Wang et al. (2025) (121)	China	Multicenter retrospective cohort	10,140 ART cycles (4,099 COVID-positive vs 6,041 controls)	Not reported	Not reported	Not reported	41.0% vs 39.7%; adjusted *p* = 0.167	32.5% vs 30.6%; adjusted *p* = 0.140	COVID-19 history did not reduce clinical pregnancy or live birth rates; embryo transfer within 30–60 days was associated with higher miscarriage risk.

aOR, adjusted odds ratio; ART, assisted reproductive technology; CI, confidence interval; COH, controlled ovarian hyperstimulation; COVID-19, coronavirus disease 2019; FET, frozen embryo transfer; ICSI, intracytoplasmic sperm injection; IVF, *in vitro* fertilization; MII, metaphase II; SARS-CoV-2, severe acute respiratory syndrome coronavirus 2.

Nevertheless, some studies indicate that disease severity and the timing of ART initiation after infection may influence selected reproductive outcomes. Dolgushina et al. reported a higher proportion of poor-quality blastocysts among women undergoing ART within 180 days of infection and suggested that moderate COVID-19 may adversely affect embryogenesis (42). Likewise, Wang et al., in the largest cohort to date involving more than 10,000 ART cycles, found no reduction in clinical pregnancy or live birth rates associated with prior COVID-19; however, embryo transfer within 30–60 days after infection was associated with an increased risk of miscarriage (121). In addition, evidence from vaccinated ART populations indicates that COVID-19 vaccination does not adversely affect ovarian stimulation outcomes, fertilization, embryo development, implantation, or ongoing pregnancy rates (122). Collectively, these findings suggest that ovarian function, fertility potential, and ART success rates are generally preserved following COVID-19, although the effects of severe disease, inflammatory burden, and treatment timing warrant further investigation in large prospective studies (118–122).

## 7 Clinical implications, knowledge gaps, and future directions

The biological evidence reviewed in Section 2 indicates that SARS-CoV-2 may affect female reproductive tissues through multiple interconnected pathways involving ACE2/TMPRSS2-mediated susceptibility, immune dysregulation, oxidative stress, endothelial injury, and neuroendocrine disturbances (1–6). However, most supporting evidence remains indirect and is derived from experimental models, transcriptomic analyses, and observational clinical studies rather than direct demonstration of persistent tissue injury *in vivo*. Consequently, the clinical significance of many proposed mechanisms, including mitochondrial dysfunction, ovarian autoimmunity, endothelial microthrombosis, and persistent inflammatory signaling, remains uncertain (4–6). Future longitudinal studies integrating tissue-based analyses with clinical and endocrine assessments are needed to distinguish transient physiological responses from clinically meaningful reproductive injury.

The heterogeneity of reproductive outcomes following COVID-19 suggests that susceptibility is influenced by interactions among inflammatory, endocrine, vascular, and individual host factors rather than by a single pathogenic mechanism (1–6). Most women experience transient or no clinically significant reproductive abnormalities, whereas others develop persistent menstrual disturbances, hormonal alterations, or reduced ovarian reserve markers (5–10). From a clinical perspective, women with severe disease, long COVID, pre-existing gynecological or metabolic disorders, obesity, autoimmune diseases, or diminished ovarian reserve may require closer follow-up because these conditions may modify inflammatory and neuroendocrine responses to SARS-CoV-2. However, current evidence remains insufficient to accurately identify high-risk individuals or support personalized risk prediction.

The available evidence indicates that menstrual disturbances represent one of the most commonly reported reproductive manifestations of COVID-19, affecting approximately 40%–60% of women during the post-infection period (1–3). Nevertheless, the highest-quality prospective studies demonstrate that cycle-length changes are generally modest and reversible, with mean increases of approximately 1–2 days that return to baseline within subsequent cycles (1). Similarly, although changes in menstrual flow, dysmenorrhea, and hormonal parameters have been reported, most studies suggest that these abnormalities are transient rather than permanent (2, 3, 8). These findings support evidence-based reassurance for most women recovering from mild-to-moderate COVID-19 while emphasizing that persistent menstrual abnormalities, repeated infections, long COVID, obesity, autoimmune diseases, or pre-existing gynecological disorders warrant individualized gynecologic and endocrine evaluation.

Current evidence suggests that female fertility potential is largely preserved following recovery from COVID-19. Most studies report no clinically meaningful deterioration in AMH, AFC, ovarian responsiveness, oocyte yield, embryo quality, clinical pregnancy rates, or ART outcomes after mild-to-moderate infection (7, 14–18). Nevertheless, conflicting findings remain regarding ovarian reserve markers, particularly AMH, where some cohorts demonstrate significant reductions associated with inflammatory burden and disease severity, whereas others report no measurable changes (5, 10, 11). Overall, available evidence does not support clinically significant impairment of fertility in most women after recovery from COVID-19. Nevertheless, women with persistent reproductive symptoms, severe infection, pre-existing ovarian disorders, or additional reproductive risk factors should receive individualized assessment and follow-up until more robust long-term evidence becomes available.

## 8 Conclusions

Accumulating evidence indicates that SARS-CoV-2 can influence female reproductive health through multiple interconnected biological pathways involving ACE2/TMPRSS2-mediated tissue susceptibility, immune dysregulation, oxidative stress, endothelial dysfunction, neuroendocrine disturbances, and potentially autoimmune mechanisms. Current evidence supports biological plausibility, but the strength of evidence varies across tissues and mechanisms. Placental involvement is supported by the strongest evidence, whereas direct ovarian infection, ovarian autoimmunity, mitochondrial dysfunction, and reproductive microthrombosis remain insufficiently validated.

Current clinical evidence suggests that menstrual disturbances are among the most frequently reported reproductive manifestations following COVID-19. Alterations in cycle length, menstrual flow, dysmenorrhea, and menstrual regularity have been documented across multiple populations. Nevertheless, the highest-quality prospective studies indicate that most menstrual changes are modest, transient, and reversible. Although hormonal alterations have been reported in some cohorts, current evidence overall supports temporary reproductive disruption rather than permanent reproductive impairment.

Evidence regarding ovarian reserve, fertility potential, and assisted reproductive technology outcomes is generally reassuring. Most studies demonstrate preserved ovarian responsiveness, embryo development, clinical pregnancy rates, and live birth outcomes following COVID-19. IVF and ICSI outcomes also appear largely unaffected by previous SARS-CoV-2 infection or COVID-19 vaccination. Collectively, these findings suggest that female reproductive capacity is generally maintained following recovery from COVID-19, although transient reproductive alterations may occur in women with severe disease or elevated inflammatory burden.

Despite substantial progress, important knowledge gaps remain. Existing evidence is limited by heterogeneous study designs, small sample sizes, inadequate representation of severe COVID-19 cases, inconsistent hormonal assessment protocols, limited longitudinal follow-up, and insufficient evaluation of live birth and long-term reproductive outcomes. Future research should prioritize large multicenter prospective cohorts integrating hormonal, imaging, inflammatory, immunological, and reproductive outcome measures together with advanced tissue-level and multi-omics analyses. Such studies will be essential for distinguishing transient physiological adaptations from clinically meaningful reproductive injury and for establishing evidence-based strategies to monitor and support reproductive recovery following SARS-CoV-2 infection.
